# Five major outcomes of digitalization: relevance of a survival personality type during COVID-19 pandemic

**DOI:** 10.3389/fpsyg.2023.1230192

**Published:** 2023-08-18

**Authors:** Yumi Hamamoto, Akio Honda, Naoki Miura, Azumi Tanabe-Ishibashi, Kentaro Oba, Ryo Ishibashi, Motoaki Sugiura

**Affiliations:** ^1^Department of Psychology, Northumbria University, Newcastle upon Tyne, United Kingdom; ^2^Department of Human Brain Science, Institute of Development, Aging and Cancer, Tohoku University, Sendai, Japan; ^3^Japan Society for the Promotion of Science, Tokyo, Japan; ^4^Department of Information Design, Faculty of Informatics, Shizuoka Institute of Science and Technology, Fukuroi, Japan; ^5^Faculty of Engineering, Tohoku Institute of Technology, Sendai, Japan; ^6^International Research Institute of Disaster Science, Tohoku University, Sendai, Japan; ^7^Center for Information and Neural Networks (CiNet), National Institute of Information and Communications Technology, Osaka, Japan

**Keywords:** digitalization, COVID-19, social, online survey, survival

## Abstract

The COVID-19 pandemic required people to adapt rapidly to the digital transformation of society for social survival, which highlighted the divide between those who can and cannot digitalize. Previous studies investigated factors promoting adaptation to digitalization; however, outcomes from adaptation to a digitalized society have not been sorted into a parsimonious model, even though there should be several multifaceted outcomes (e.g., usefulness, economic profit, and social outcome), each of which is promoted by different factors. If the effects of individual background factors can be revealed, including the technical-environment and survival-relevant personality in relation to each outcome, it would help in the creation of a society where more people play an active role by adapting to digitalization. This study aimed to construct such a model by identifying major outcomes gained in a digitalized society and investigating individual factors that contribute to the degree of gain of each of these outcomes. Five dimensions were identified by online surveys and factor analysis: Socialization (outcomes derived from new social connections created online), Space–time (freedom from time and space constraints), Economics (monetary outcome by using digital services), and Information (ease and amount of acquisition of information) were the positive outcomes, whereas Loneliness (feelings of not being able to keep up with digitization) was identified as a negative outcome. We determined that technical-environmental factors (e.g., familiarity with digital techniques and the amount of money that can be used for digitalization) facilitated gain in four positive outcomes. Notably, leadership and conscientiousness facilitated the Socialization gain while etiquette suppressed it. These factors’ effects would reflect the importance of a personality trait prioritizing construction and maintenance of social relationships. This study implies that material outcomes (i.e., Space–time, Economics, and Information) are promoted by technical-environmental support, whereas social outcomes may additionally require motivation and a positive attitude for purposeful social engagement.

## 1. Introduction

Digitalization technologies have permeated society leading to enormous changes. For example, applications for various services, including administrative services, can now be made online. Individuals gain various positive outcomes by working in a digitalized society, such as saving commuting time by working remotely (Collins and Wellman, 2010; Alam et al., 2014). However, individuals also experience negative outcomes in a digitalized society, such as decreased physical activity (Rajani and Chandio, 2004; Slonje et al., 2013; Alam et al., 2014). Previous studies included a broad range of outcomes. Several studies referred to the same outcomes as either positive or negative according to their research interest (e.g., online communication tools increase the total amount of communication, but tend to decrease in-person communication) (Collins and Wellman, 2010; Elhai et al., 2016). Relatively indirect outcomes, such as a decrease in the amount of learning time because of the Internet, have also been reported (Rajani and Chandio, 2004). Other studies have addressed specific outcomes in the workplace, such as employees’ accessibility to documents via Cloud computing (Diller et al., 2020; Yang et al., 2022).

Previous studies have reported that various positive outcomes are classified into material and immaterial outcomes. Material outcomes are those related to benefits from earning money, saving time, and obtaining information (Rajani and Chandio, 2004; Collins and Wellman, 2010; Alam et al., 2014; Scheerder et al., 2020). In contrast, several studies have referred to two immaterial outcomes: skill-building and social interaction (Rajani and Chandio, 2004; Alam et al., 2014; Scheerder et al., 2020). Material outcomes, such as earning money (Scheerder et al., 2020), saving time (Collins and Wellman, 2010), and gaining information (Rajani and Chandio, 2004; Alam et al., 2014), can be easily gained in everyday life, whereas social immaterial outcomes cannot. Social immaterial outcomes require individuals to adapt to a digitalized society and interact with others; thus, these outcomes would be advanced compared with other outcomes. Moreover, gaining social immaterial outcomes is important for maintaining a mutual connection with others, particularly under the rapid digitalization caused by the COVID-19 pandemic.

The importance of adapting to a digitalized society has been discussed in various fields. Previous studies have reported that those who use the Internet more tend to experience positive outcomes compared with those who use the Internet less (Nie and Erbring, 2001; Rajani and Chandio, 2004). For example, individuals who work remotely have much more leisure time than individuals who commute every day. Moreover, how much a person adapts to a digitalized society is linked to social disparity; accessibility to the Internet is related to educational equity (Gorski, 2005), mental health (Ennis et al., 2012), and COVID-19 mortality rates (IDEA, 2022; Li, 2022).

Achieving a society in which people adapt to digitalization and gain positive outcomes has attracted attention. For instance, the relationships between adaptation to digital technologies and personal environmental factors have been investigated. Socioeconomic status affects digitalization because it affects adopting some technologies, such as accessibility to the Internet and digital devices (Beilock and Dimitrova, 2003; Quibria et al., 2003; Kraemer et al., 2005; Billon et al., 2010), which are needed to live in a digitalized society. Previous studies have reported that individuals with higher education tend to have more positive outcomes derived from digitalization (Al-Zahrani, 2015; Scheerder et al., 2020). Moreover, digitalization has been rapidly facilitated in several countries in the wake of the COVID-19 pandemic. Several digitalization-related technical–environmental factors, such as education and accessibility to digital technologies, can be improved by public assistance and intervention; thus, central and local governments in many countries are trying to create an environment that enables more individuals to adapt to a digitalized society and gain more positive outcomes (e.g., the Digital Agency was established by the Japanese government in 2021).

It is also important to investigate relationships between digitalization and personality factors to reveal what kind of people can and cannot adapt to a digitalized society, regardless of the environment. Previous studies have investigated relationships between the Big Five personality traits and digitalization (Diller et al., 2020; Maran et al., 2022). Those studies reported that individuals with higher extraversion and openness and lower neuroticism tended to promote digitalization.

However, there are two major issues in revealing the kinds of people who gain positive outcomes in a digitalized society. First, there is no established parsimonious multidimensional model, despite the multifaceted nature of digitalization outcomes. A common concept of outcomes in a digitalized society has not been established and previous studies investigated specific outcomes according to the researchers’ interests. This lack of an established multidimensional model has made it difficult to compare previous findings related to factors affecting outcomes in digitalization. Establishing parsimonious models would allow us to integrate findings from different studies. Second, the factors affecting digitization outcomes have not been fully explored. There is also no established parsimonious model for background environmental factors. Previous studies investigated the effects of various specific environmental factors on digitalization (e.g., socioeconomic status, Internet accessibility, and educational background) according to the researchers’ interests (Gorski, 2005; Al-Zahrani, 2015; Scheerder et al., 2020). The lack of an established multidimensional model makes it difficult to investigate comprehensively the relationships between outcomes and background environmental factors. Personality traits related to adaptation to environmental change have not been explored. Previous studies investigating relationships between personality factors and digitalization focused only on relationships between the Big Five personality traits and digitalization (Diller et al., 2020; Maran et al., 2022). As digitalization has been rapidly facilitated to adapt to society during the COVID-19 pandemic and those who cannot adapt to a digitalized society suffer disadvantages (Gorski, 2005; Ennis et al., 2012; IDEA, 2022; Li, 2022), the ability to adapt to digitalization is possibly related to the ability to survive a disaster. The relationship between survival personality traits and adaptation to a digitalized society is of interest.

In this study, we aimed to reveal what kind of people adapt to a digitalized society and gain more positive outcomes, while avoiding negative outcomes. Thus, we first established a parsimonious model of digitalization outcomes. We conducted a free-description survey and investigated what people perceive as positive and negative outcomes in a digitalized society. Candidate outcomes were determined from the descriptions. Then, factor analysis was performed to establish a questionnaire of digitalization outcomes (i.e., digitalization outcome inventory). Second, we investigated relationships among the outcomes and background factors. We sorted the digitalization-related environment and attitude factors in the same way as we determined the outcomes. We asked participants about the characteristics of those who would tend to gain such outcomes easily, and determined candidate background factors to create a questionnaire of digitalization background factors (i.e., digitalization background environment and attitude questionnaire). Considering disaster survival characteristics in relation to the rapid digitalization stimulated by COVID-19, we also examined survival-related personality traits using the Power to Live questionnaire (Sugiura et al., 2015), which evaluates eight survival-related personality traits. To examine other general personality traits, we examined the participants’ Big Five personality traits (Barrick and Mount, 1991). Using these factors as dependent values, we performed hierarchical multiple regression analysis to reveal relationships between the outcomes and the background factors. Each outcome from the digitalization outcome inventory was an objective variable predicted by individual characteristics. We used the participants’ demographic data and scores from the questionnaires as dependent variables.

Based on the parsimonious model of outcomes, we expected to find a range of outcomes from a simple material outcome, such as the usefulness of using the Internet, to an advanced social immaterial outcome, such as the feeling of expanding one’s social connection and world. It was expected that different background factors would affect the gaining of each outcome. We expected that technical-environmental factors, such as socioeconomic status, would affect gaining any outcomes and that gaining advanced social immaterial outcomes would enhance some survival-related personality traits on the Power to Live questionnaire.

## 2. Materials and methods

We conducted a two-phase online survey in this study. The first phase of the online survey was a free-description survey and we collected candidate descriptions of the outcome model and those of the background environment and the attitude model. In the second phase, participants scored the self-relevance of the candidate descriptions for each model, and we collected demographic data and questionnaire scores for the Power to Live and Big Five questionnaires. We conducted factor analysis using candidate item scores from the outcome model and established a parsimonious multidimensional model of digitalization outcomes. Similarly, we established a background environmental and attitude model using candidate item scores of the tentative model. Finally, we conducted hierarchical multiple regression analysis for each dimension of the outcome models; independent values were the scores of each dimension of the outcome model and the dependent values were the participants’ demographic data and questionnaire scores, including the digitalization background environment and the Big Five and Power to Live questionnaires.

### 2.1. Ethical statement

This study was approved by the Ethical Committee of the International Research Institute of Disaster Science, Tohoku University, Japan (2020-021 and 2022-012). Informed consent was obtained from all participants following the tenets of the Declaration of Helsinki.

### 2.2. Collecting candidate descriptions of the models

#### 2.2.1. Participants

The survey was conducted online by Cross Marketing Ink (Tokyo, Japan) in December between 4 and 7 December 2020. The survey companies emailed an advertisement of the survey to the company’s registered pool of possible online crowd workers living in any of the 47 prefectures of Japan. The responders participated in exchange for an online voucher/shopping points. To recruit equally, regardless of sex and age, we created 12 groups according to sex and age; males or females in their 20s, 30s, 40s, 50s, 60s, and 70s and older groups (two sexes × six age ranges). Each group included 20 participants; thus, 240 people (males = 120) participated in our online survey. Applicants who answered with meaningless responses (e.g., ‘None’, ‘I cannot think of anything’, and so on) were not included in this 240-participant dataset. Participants’ mean age ± SD (standard deviation) was 49.8 ± 16.4.

#### 2.2.2. Design of the online survey

We asked the following three questions to collect candidate descriptions of the outcome model: “What digital technologies do people adapting to a digitalized society use, how do they use those technologies, and what outcomes do they gain?,” “What are some of the situations where people cannot adapt to a digitalized society; why does this occur, and at what disadvantage are they?,” and “Why are people poor at in-person communication, what situations tend to be promote this, and at what disadvantage are they?”

We also asked the following three questions to collect candidate descriptions of the background environment and the attitude model: “What are the characteristics of people who adapt to a digitalized society?,” “What are the characteristics of people who cannot adapt to a digitalized society?,” and “What are the characteristics of people who are poor at in-person communication?”

#### 2.2.3. Determining the candidate items

To determined candidate items for the digitalization outcome inventory and the digitalization background questionnaires, we adopted the label making and the label grouping steps of the KJ method (Scupin, 1997). That is to say, we first changed each description that was gained by the free-description survey to contain only one idea (e.g., we changed “I can save time and money” to “I can save time” and “I can save money”). Then, we intuitively created categories of sentences with similar descriptive content with corresponding headlines. Finally, five or more sentences from each category were chosen as candidate items for the questionnaires. If there were fewer than five original descriptive sentences in a category, all of the original sentences were chosen as candidate items.

Sixty-one candidate items were prepared for the digitalization outcome inventory, based on nine tentative categories. Twenty-six candidate items were prepared for the digitalization background environment and attitude questionnaires based on five tentative categories.

### 2.3. Establishing the models and investigating relationships between the outcomes and background factors

#### 2.3.1. Participants

The survey was conducted online by Neo Marketing (Tokyo, Japan) between 15 and 20 July 2022. The survey companies emailed an advertisement of the survey to the company’s registered pool of possible online crowd workers living in any of the 47 prefectures of Japan. The responders participated in exchange for an online voucher/shopping points. We recruited the participants considering sex and age. We created 12 groups according to sex and age: males or females in their 20s, 30s, 40s, 50s, 60s, and 70s and older groups (2 sexes × 6 age ranges). Each group included 100 participants; thus, 1,200 people (males = 600) participated in the online survey. Participants’ sociodemographic data were described in Table 1.

**Table 1:** Participants’ sociodemographic data (the second online survey).

	Mean (SD)
Age	49.8 (16.8)
	Frequency (percentage)
Annual income	
0–2,000,000 yen	618 (51.5)
2,000,000 – 3,990,000 yen	318 (26.5)
4,000,000 – 5,990,000 yen	147 (12.3)
6,000,000 – 7,990,000 yen	64 (5.33)
8,000,000 – 9,990,000 yen	24 (2.00)
10,000,000 – 11,990,000 yen	10 (0.83)
12,000,000 – 13,990,000 yen	7 (0.58)
over 14,000,000 yen	12 (1.00)
Educational background	
Junior high school graduate	35 (2.92)
High school graduate	359 (29.9)
College graduate	219 (18.3)
University graduate	509 (42.4)
Master course graduate	68 (5.67)
Doctoral course graduate	10 (0.83)

Age was the chronological age and its mean was described. Annual income and educational background were analyzed as categorical data, and frequency of each variable was described. Annual income was chosen from eight options (1 = 0–2,000,000 yen, 2 = 2,000,000 – 3,990,000 yen; 3 = 4,000,000 – 5,990,000 yen; 4 = 6,000,000 – 7,990,000 yen; 5 = 8,000,000 – 9,990,000 yen; 6 = 10,000,000 – 11,990,000 yen; 7 = 12,000,000 – 13,990,000 yen; 8 = over 14,000,000 yen). Educational background was chosen from six options (1 = junior high school graduate, 2 = high school graduate; 3 = college graduate; 4 = university graduate; 5 = master course graduate; 6 = doctoral course graduate).

SD, standard deviation.

#### 2.3.2. Indices examined

Participants answered the candidate items on the digitalization outcome inventory and the digitalization background environment and attitude questionnaires using a 6-point Likert scale (from 0: not applicable at all to 5: very applicable). In addition to these candidate items, we examined participants’ sociodemographic data (age, annual income and educational background), the Big Five personality traits and survival-related personality traits, and the degree of Internet usage. Age was the chronological age and participants answered their annual income from eight options (from 0 yen to over 14,000,000 yen) and educational background from six options (from junior high school graduate to doctoral course graduate). To evaluate participants’ survival-related personality traits, we used the Japanese version of the Power to Live questionnaire (Sugiura et al., 2015). This questionnaire is composed of 34 items originally written in Japanese. The 34 items are classified into eight dimensions: leadership (five items), problem-solving (five items), altruism (five items), stubbornness (five items), etiquette (three items), emotion regulation (four items), self-transcendence (four items), and active well-being (three items). To evaluate participants’ Big Five personality traits, we used the Japanese version of the Ten-Item Personality Inventory (Gosling et al., 2003; Oshio et al., 2012). This inventory is composed of ten items; two items are used to evaluate the Big Five personality traits of extraversion, agreeableness, conscientiousness, neuroticism, and openness. The degree of Internet usage was examined as the extent to which each participant used 17 various online services in daily life (Ministry of Internal Affairs and Communications, 2021). Participants rated the frequency of usage on a 6-point Likert scale (from 0: never use to 5: use frequently).

#### 2.3.3. Factor analysis to determine the structure of the questionnaires

We confirmed the appropriateness of the data for exploratory factor analysis by performing the Kaiser-Meyer-Olkin test. Then, the number of factors was examined using a scree plot, parallel analysis (Horn, 1965), minimum average partial correlation (Velicer, 1976), and the Bayesian information criterion (Schwarz, 1978). We used the minimum residuals method with the oblimin rotation. Items with loading 0.3 were excluded, and we repeated the analysis until all items met the criteria. Cronbach’s was calculated to evaluate the internal construct validity for each factor.

#### 2.3.4. Analysis to investigate relationships between adaptation to digitalization and background factors

Items for each outcome and background factor were summed to determine the outcome and background factor scores. Items with negative loadings were reverse-coded. Then, all data, including scores from the digitalization outcome inventory and the digitalization background environment and attitude questionnaire, were standardized.

The hierarchical multiple regression was comprised of three steps. We adopted the forced entry method for step 1; demographic data (age, personal income, and educational background) were entered into the model. The forward-backward stepwise method was adapted for steps 2 and 3. Scores of each factor from the digitalization background environment and attitude questionnaire were examined in step 2. Finally, scores of each factor from the Big Five personality traits and the Power to Live instruments were examined in step 3.

After developing multiple regression models for each factor from the digitalization outcome inventory, the significance of each standardized regression coefficient was evaluated according to the effect size (Selya et al., 2012; Ding et al., 2022). We examined Cohen’s *f* ^2^; Cohen’s *f*_A_ was the effect size of a model including all regressors, Cohen’s *f*_B_ was the effect size of a model excluding one regressor (e.g., regressor X), and Cohen’s *f*_B/A_^2^ was the effect size of a specific regressor (i.e., regressor X). Previous studies classified the effect size as small 0.020–0.085, medium 0.086–0.250, and large >0.250 (Selya et al., 2012; Taylor et al., 2020). Thus, the significance threshold in this study was set at Cohen’s *f*_B/A_^2^ ≥ 0.02.

## 3. Results

### 3.1. Factor analysis of digitalization outcome inventory

The factor analysis identified the following seven dimensions: Socialization (social outcome from online communication), Space–time (freedom from time and space constraints), Loneliness (feelings of not being able to keep up with digitization), Economic (monetary outcome gained using digital services), Preference (preference toward online communication), Communication decrease (decrease in in-person communication), and Information (ease and amount of acquisition of information). The descriptions of all items, loadings, and Cronbach’s α values of each factor are listed in Table 2.

**Table 2:** Details of the digitalization benefit inventory.

Description	Factor loadings							*α*
		F1	F2	F3	F4	F5	F6	F7
**F1: Socialization**								**0.94**
I increased my presence by promoting myself on the Internet	0.72	0.06	0.10	0.03	0.08	−0.07	0.00	
I gained new clients and jobs through Internet activities	0.68	0.13	0.06	0.09	−0.04	−0.08	−0.10	
I built relationships online with people I would never meet in my everyday life	0.67	−0.04	0.01	0.03	0.15	0.18	0.04	
Business opportunities were created that had never existed before	0.65	0.17	0.09	0.05	−0.01	−0.10	−0.09	
I felt I benefited from using the Internet for networking	0.65	0.03	0.11	0.03	0.10	0.06	0.04	
Internet-based activities satisfied my need for approval	0.53	0.11	0.03	0.10	0.12	0.02	0.12	
I felt my ability to read the thoughts of others increased	0.51	0.16	0.14	0.00	0.10	−0.02	0.14	
I felt that the information I put out on the Internet might have an impact that could not be ignored	0.49	−0.03	0.19	0.10	0.04	0.04	0.08	
I felt connected to people with whom it is difficult to meet face to face	0.49	0.11	0.11	0.00	0.07	0.18	0.08	
I used Internet-based advertising and promotion	0.44	0.06	0.07	0.15	−0.01	0.07	0.11	
Online interaction became the main focus	0.43	0.09	0.00	0.05	0.22	0.35	−0.10	
I am used to interacting online	0.41	0.18	−0.19	0.09	0.26	0.33	−0.03	
I found it easier to speak up online	0.40	0.03	0.05	0.05	0.40	0.09	0.10	
**F2: Space–time**								**0.93**
I have more time for myself	−0.08	0.75	0.10	0.01	−0.04	0.10	0.02	
I set my own leisure time	−0.04	0.74	−0.03	0.02	−0.03	0.05	0.12	
My physical load has been reduced	−0.05	0.68	0.05	0.02	0.18	0.08	0.00	
I could do a lot of other work in my free time	0.12	0.67	0.02	0.09	−0.04	0.02	0.06	
I saved time	0.05	0.66	0.00	0.13	0.04	0.01	0.04	
My mental state was stable	0.02	0.63	−0.04	−0.01	0.18	−0.09	0.16	
Remote work has given me more time in my life	0.32	0.60	0.01	0.03	−0.05	0.04	−0.29	
My mental load has decreased	−0.02	0.58	0.04	0.06	0.32	−0.06	0.04	
The number of opportunities to commute to work and school has decreased	0.17	0.53	0.08	−0.02	−0.01	0.12	−0.31	
I can work efficiently anytime, anywhere	0.30	0.47	−0.05	0.14	−0.01	0.03	0.07	
I can quickly solve problems as they arise	0.23	0.38	−0.02	0.18	0.00	−0.04	0.20	
**F3: Loneliness**								**0.90**
I feel left out by others	0.03	0.02	0.76	−0.02	−0.05	−0.02	−0.04	
I feel my ability to read the thoughts of others is declining	0.02	0.06	0.68	0.02	0.06	0.07	0.02	
I feel isolated	0.00	−0.06	0.66	0.05	−0.01	0.14	−0.11	
When I talk to others in person, I feel I do not get a good sense of trust from them	0.17	0.02	0.65	0.01	0.13	−0.01	−0.03	
I feel I cannot not keep up with digitalized society	−0.10	−0.01	0.65	−0.13	−0.11	−0.06	0.13	
When I talk to others in person, I feel I do not speak well	−0.06	0.02	0.65	0.09	0.28	−0.04	−0.06	
I find it difficult to understand the ideas and stories of those who can keep up with new technologies, information, and trends	0.06	0.02	0.63	0.03	−0.02	0.03	0.14	
I feel it is more difficult to build relationships in face-to-face interactions	0.06	0.02	0.56	0.04	0.00	0.27	−0.01	
I feel as if they would not seriously help me when I needed it in face-to-face interactions	0.13	−0.01	0.54	0.05	0.03	0.11	0.08	
I feel I could miss the trend and lose my job opportunities	0.36	0.01	0.54	0.03	−0.05	−0.06	−0.05	
I feel my means of communication are limited	0.06	0.04	0.40	0.01	−0.14	0.38	0.10	
**F4: Economic**								**0.88**
Cashless payment allows me to save money by using points and rewards compared to a cash payment	−0.04	−0.06	0.01	0.86	−0.02	−0.06	0.01	
I use cashless payment	−0.08	−0.05	−0.01	0.80	−0.03	0.05	−0.08	
I shop without a wallet	0.23	0.01	0.04	0.50	−0.02	−0.05	−0.08	
I compare products on the Internet	−0.08	0.13	0.00	0.50	0.07	0.12	0.17	
I use discount services, etc., which can only be applied for *via* the Internet	0.21	0.07	0.04	0.49	0.01	−0.02	0.10	
I purchase goods or services at a great price (e.g., a big sale or discount)	0.17	0.11	0.03	0.47	0.01	−0.01	0.12	
I complete various procedures (e.g., smartphone contracts, grant applications, etc.) quickly through the online application process	0.19	0.14	−0.03	0.46	0.03	0.07	0.03	
I use the Internet to gather and disseminate information	−0.10	0.13	−0.05	0.40	0.08	0.25	0.21	
I perform routine tasks (e.g., shopping, controlling appliances) from my computer or smartphone without taking up time	0.11	0.29	−0.01	0.39	0.08	0.04	0.04	
**F5: Preference**								**0.80**
I feel that online interactions are preferable to in-person meetings	0.24	0.18	0.04	0.02	0.56	−0.01	0.01	
I feel it is easier to interact with others online	0.38	0.08	0.01	0.04	0.52	0.07	0.09	
I find it bothersome to meet others and talk to them face to face	−0.14	0.18	0.32	0.13	0.51	−0.09	−0.04	
**F6: Communication decrease**								**0.75**
Opportunities to feel the human warmth that comes from actually meeting other people has decreased	−0.04	0.12	0.35	0.05	−0.21	0.51	0.14	
Opportunities to meet and talk with people have decreased	−0.19	0.17	0.19	0.17	0.04	0.50	−0.03	
Outside of online interactions, friendships have narrowed	0.06	0.00	0.32	0.09	0.12	0.39	−0.04	
**F7: Information**								**0.76**
I was able to learn detailed information that I could not find on TV or in magazines	0.25	0.19	0.08	0.12	−0.01	0.07	0.43	
I was able to find out what I wanted to know right away	−0.01	0.20	−0.03	0.33	0.04	0.06	0.39	
I was able to get local information quickly	0.33	0.17	0.07	0.15	0.01	0.02	0.35	

Descriptions of each item, factor loadings, and the Cronbach’s *α* value of each factor are described.

Preference and Communication decrease were not related to outcomes of a digitalized society. The items in preference did not describe the outcomes, and the items in communication decrease were related to restrictions under the COVID-19 pandemic rather than the effects of a digitalized society. Thus, five dimensions were identified as factors of the digitalization outcome inventory: four positive dimensions (Socialization, Space–time, Economic, and Information) and one negative dimension (Loneliness).

### 3.2. Contributing factors of each outcome

#### 3.2.1. Factor analysis of digitalization background environment and attitude questionnaire

Factor analysis identified the following five factors: unfamiliarity with digital technologies (feeling unfamiliar and distrustful of digital technologies), analog preference (preference toward analog things), budget for digitalization (the amount of money that can be used for digitalization), conservativeness (preference for old things), and digital technology-friendly environment (an environment that allowed a participant to access digital technologies frequently). Descriptions of all of the items, loadings, and Cronbach’s α values of the factors are listed in Table 3.

**Table 3:** Details of the digitalization background environment and attitude questionnaire.

Description	Factor loadings					*α*
		F1	F2	F3	F4	F5
**F1: unfamiliarity with digital technologies**						**0.87**
I do not know how to use the Internet	0.85	−0.04	0.02	−0.01	−0.03	
I have not acquired any knowledge about the Internet	0.79	0.01	0.04	0.02	−0.11	
I think the Internet is difficult	0.77	0.07	−0.02	−0.05	−0.03	
I do not know what digitalization is	0.70	−0.06	−0.05	0.06	0.16	
I have negative feelings about digital technology	0.51	0.21	−0.08	0.10	0.09	
I am not as energized to keep up with new things as before	0.45	0.12	−0.07	0.12	−0.04	
**F2: analog preference**						**0.82**
I think information obtained in person is more trustworthy than information obtained from the Internet	−0.05	0.78	0.01	−0.04	0.02	
I trust what is on paper more	0.10	0.63	−0.01	0.07	0.00	
I value face-to-face communication	−0.05	0.62	0.11	0.06	−0.01	
I only trust what I can see	0.11	0.61	−0.09	0.07	0.03	
I prefer analog things (newspapers, records, paper books, etc. that are not digitized)	0.04	0.47	0.02	0.18	−0.08	
I think the Internet is dangerous	0.28	0.37	−0.13	0.00	0.02	
**F3: budget for digitalization**						**0.91**
I can afford to purchase a computer and other equipment	−0.01	0.02	0.93	0.00	−0.05	
I have the financial savvy to spend money on technologies	0.04	−0.07	0.85	0.02	0.08	
I have enough money to develop an environment for using the Internet	−0.04	0.05	0.82	−0.02	0.02	
**F4: conservativeness**						**0.76**
I have an old-fashioned way of thinking and values	0.00	−0.05	−0.01	0.89	0.01	
I value old customs	−0.02	0.16	0.05	0.68	−0.01	
I am relatively conservative	0.05	−0.01	−0.06	0.52	−0.05	
**F5: digital technology-friendly environment**						**0.53**
I grew up in an environment where the Internet and other information and communication technologies were all around me	−0.07	0.07	0.09	0.01	0.64	
I have to be comfortable with digital technology (remote meetings and online classes) due to the nature of my job duties	0.09	0.00	0.12	0.01	0.50	
I did not receive any education about the Internet*	0.20	0.16	0.11	0.10	−0.41	

Descriptions of each item, factor loadings, and Cronbach’s α of each factor were described. An asterisk indicates an inverted scale.

#### 3.2.2. Correlations with the digitalization outcome inventory

Correlations between the five dimensions from the digitalization outcome inventory and five factors from the digitalization background environment and attitude questionnaire, the five factors from the Big Five personality traits inventory, the eight factors from the Power to Live questionnaire, three demographic factors (age, personal income, and educational background), and the degree of Internet usage are described in Table 4.

**Table 4:** Correlation coefficients between factors from the digitalization outcome inventory and other factors.

	Socialization	Space–time	Loneliness	Economic	Information
Demographic data					
Age	−0.26*	−0.16*	−0.15*	−0.14*	−0.04
Personal income	0.13*	0.13*	−0.04	0.11*	0.01
Educational background	0.14*	0.13*	0.01	0.12*	0.02
Digitalization background environment and attitude questionnaire					
Unfamiliarity with digital technologies	−0.11*	−0.17*	0.36*	−0.22*	−0.11*
Analog preference	−0.09	−0.10*	0.32*	−0.13*	−0.01
Budget for digitalization	0.24*	0.30*	−0.01	0.32*	0.25*
Conservativeness	−0.07	−0.06	0.21*	−0.09	0.02
Digital technology-friendly environment	0.35*	0.34*	0.26*	0.26*	0.24*
Power to live					
Leadership	0.31*	0.22*	0.09	0.19*	0.20*
Problem-solving	0.23*	0.25*	0.03	0.28*	0.28*
Altruism	0.23*	0.17*	0.19*	0.19*	0.21*
Stubbornness	0.18*	0.17*	0.05	0.16*	0.20*
Etiquette	0.00	0.11*	−0.02	0.19*	0.18*
Emotional regulation	0.20*	0.22*	0.02	0.21*	0.21*
Self-transcendence	0.22*	0.23*	0.08	0.25*	0.25*
Active well-being	0.35*	0.34*	0.11*	0.30*	0.32*
Big five					
Extraversion	0.21*	0.19*	0.11*	0.17*	0.18*
Agreeableness	0.23*	0.15*	0.23*	0.14*	0.15*
Conscientiousness	0.20*	0.21*	0.25*	0.18*	0.19*
Neuroticism	0.10*	0.13*	0.16*	0.15*	0.16*
Openness	0.16*	0.16*	0.22*	0.21*	0.19*
Actual Internet usage					
Internet usage	0.49*	0.44*	0.18*	0.61*	0.39*

Correlation coefficients from simple correlation analysis are described. Asterisks indicate correlation coefficients above the significant threshold (*r* ≥ 0.1) for reference purposes. This significant threshold was determined based on a previous study classifying that |*r*| ≥ 0.1 has the least small effect size (Cohen, 1992).

#### 3.2.3. Multiple linear regression model for the digitalization outcome inventory

We investigated which factors influenced the five dimensions from the digitalization outcome inventory. Multiple linear regression analysis showed that different technical-environment and personality factors influenced the digitalization outcomes. Socialization was promoted by a digital technology-friendly environment (*f*_B/A_^2^ = 0.09), conscientiousness (*f*_B/A_^2^ = 0.03), leadership (*f*_B/A_^2^ = 0.03), and active well-being (*f*_B/A_^2^ = 0.03) while it was suppressed by age (*f*_B/A_^2^ = 0.03) and etiquette (*f*_B/A_^2^ = 0.02). Space–time was promoted by the budget for digitalization (*f*_B/A_^2^ = 0.03), a digital technology-friendly environment (*f*_B/A_^2^ = 0.08), and active well-being (*f*_B/A_^2^ = 0.04). Loneliness was promoted by unfamiliarity with digital technologies (*f*_B/A_^2^ = 0.09), analog preference (*f*_B/A_^2^ = 0.03), a digital technology-friendly environment (*f*_B/A_^2^ = 0.04), openness (*f*_B/A_^2^ = 0.02), and active well-being (*f*_B/A_^2^ = 0.02). Economics was promoted by the budget for digitalization (*f*_B/A_^2^ = 0.04) and openness (*f*_B/A_^2^ = 0.03) while it was suppressed by age (*f*_B/A_^2^ = 0.02). Information was promoted by the budget for digitalization (*f*_B/A_^2^ = 0.02), a digital technology-friendly environment (*f*_B/A_^2^ = 0.02), openness (*f*_B/A_^2^ = 0.02), and active well-being (*f*_B/A_^2^ = 0.02). We described standardized regression coefficients of the multiple regression models, which were identified by hierarchical multiple regression analysis and Cohen’s *f*_B/A_^2^ effect sizes of each standardized regression coefficient in Table 5. Standardized regression coefficients and the coefficient of determination of each step are listed in Supplementary Table 1.

**Table 5:** Standardized regression coefficients and Cohen’s *f* value for the standardized regression coefficients.

	Socialization		Space–time		Loneliness		Economic		Information	
	*β*	*f*_B/A_^2^	*β*	*f*_B/A_^2^	*β*	*f*_B/A_^2^	*β*	*f*_B/A_^2^	*β*	*f*_B/A_^2^
Step 1										
Age	**−2.27**	**0.03**	−1.09	0.01	−1.04	0.01	**−1.61**	**0.02**	−0.09	0.00
Personal income	−1.02	0.01	−0.62	0.00	−0.42	0.00	−0.51	0.00	−0.43	0.01
Educational background	0.05	0.00	0.04	0.00	0.10	0.00	0.16	0.00	−0.21	0.00
Step 2										
Unfamiliarity with digital technologies	0.09	0.00			**0.60**	**0.09**	−0.18	0.01		
Analog preference					**0.34**	**0.03**				
Budget for digitalization	0.30	0.01	**0.51**	**0.03**			**0.52**	**0.04**	**0.13**	**0.02**
Conservativeness										
Digital technology-friendly environment	**1.18**	**0.09**	**1.09**	**0.08**	**0.67**	**0.04**	0.38	0.01	**0.15**	**0.02**
Step 3										
Leadership	**0.57**	**0.03**								
Problem-solving									0.06	0.00
Altruism					0.23	0.01				
Stubbornness					−0.10	0.00				
Etiquette	**−0.73**	**0.02**			−0.27	0.00	0.40	0.01		
Emotional regulation					−0.20	0.00				
Self-transcendence	0.20	0.00								
Active well-being	**0.73**	**0.02**	**0.72**	**0.04**	**0.59**	**0.02**	0.31	0.01	**0.19**	**0.02**
Extraversion										
Agreeableness	0.55	0.00								
Conscientiousness	**0.53**	**0.03**	0.75	0.01	0.77	0.01	0.41	0.00	0.17	0.00
Neuroticism			0.48	0.00			0.46	0.00	0.17	0.00
Openness	0.51	0.01	0.57	0.01	**0.93**	**0.02**	**0.97**	**0.03**	**0.29**	**0.02**

Only standardized regression coefficients in the multiple regression models identified by hierarchical multiple regression analysis are described. The significance threshold was f_B/A_^2^ ≥ 0.02. The significant standardized regression coefficients are shown in bold.

## 4. Discussion

To reveal types of people who can adapt to a digitalized society, gain more positive outcomes, and avoid experiencing negative outcomes, this study aimed to establish a parsimonious multidimensional model of digitalization outcomes. Then, we investigated relationships between outcomes and background factors. First, we identified the parsimonious model of digitalization outcomes composed of five dimensions: Socialization, Space–time, loneliness, Economics, and Information. Digitalization-related background environment and attitude factors were also sorted and we identified a five-dimension model: unfamiliarity with digital technologies, analog preference, budget for digitalization, conservativeness, and a digital technology-friendly environment. Hierarchical multiple regression analysis showed that each outcome was explained by different background factors. Notably, Socialization, an immaterial positive outcome, was specifically affected by leadership, etiquette, and conscientiousness. As expected, survival personality traits influenced Socialization. These results imply that specific background factors may be expected to intervene according to the target outcomes, to allow people to gain positive digitalization outcomes.

### 4.1. General discussion regarding identified outcomes and background factors

The digitalization outcome model should be parsimonious. Identified outcomes have been referred to by previous studies. Socialization has been referred to as increasing communication and educating oneself (Rajani and Chandio, 2004; Al-Zahrani, 2015; Scheerder et al., 2020). Space–time has been referred to in the context of saving time (Nie and Erbring, 2001; Coyle, 2006; Yang et al., 2022) and rural quality of life (Collins and Wellman, 2010). Loneliness has been referred to as a decrease in physical communication (Nie and Erbring, 2001; Alam et al., 2014). Economics has been referred to as a context of earning money using the Internet (Scheerder et al., 2020) and saving money (Coyle, 2006). Information has been referred to as a tool to collect information quickly, even if used for academic purposes (Rajani and Chandio, 2004; Alam et al., 2014; Scheerder et al., 2020). Thus, we established a parsimonious multidimensional model of digitalization outcomes integrating previous research.

Among the five identified outcomes, we considered Socialization to be particularly important in the context of its relationship to self-actualization, unlike the other material outcomes. Our results imply that the contribution of social interaction to self-actualization is the core feature of items in Socialization. We assumed there would be two social immaterial outcomes when candidate items of the digitalization outcome inventory were determined. Some items regarding social immaterial outcomes would be related to self-actualization by online communication (e.g., “I increased my presence by promoting myself on the Internet,” “I used Internet-based advertising and promotion”) and other items regarding social immaterial outcomes would be related to mutual communication (e.g., “I built relationships online with people I would never meet in my everyday life,” “I felt I benefited from using the Internet for networking”). Our finding that these items were synthesized in one category by the factor analysis implies that Socialization is an outcome achieved by self-actualization using online communication rather than an outcome achieved by mere online communication as an alternative to telephone or email. Outcomes related to skill-building, which was assumed to be an immaterial outcome in some studies (Rajani and Chandio, 2004; Alam et al., 2014; Scheerder et al., 2020), are related to self-esteem in Maslow’s hierarchy of needs (Maslow, 1954). Considering our results and the previous implication that Maslow’s theory is also applied in a digitalized society (Oomen-Early and Murphy, 2009; Shipunova et al., 2019), the core feature of immaterial outcomes would be self-actualization by online social interaction.

### 4.2. General discussion regarding the identified background environment and attitude factors

Similar to the identified outcomes, we established a parsimonious model of the background environment and attitude factors. The factors of the digitalization background environment and attitude questionnaire were referred to in several previous studies. Unfamiliarity with digital technologies has been referred to as a factor in avoiding digitalization (Yang et al., 2023). Analog preference conservativeness has been indirectly referred to as an effect of openness to digitalization (Diller et al., 2020; Maran et al., 2022). Budget for digitalization has been referred to as personal or household income factors influencing digitalization (Beilock and Dimitrova, 2003; Quibria et al., 2003; Kraemer et al., 2005; Billon et al., 2010). The digital technology-friendly environment has been discussed in the context of a relationship with socioeconomic status and telecommunication infrastructure (Quibria et al., 2003; Billon et al., 2010). Thus, our background environment and attitude model should be a parsimonious model, so the digitalization background environment and attitude questionnaire was used to evaluate digitalization-related characteristics.

### 4.3. Background factors of positive outcomes

Several common background factors influenced gaining positive outcomes, as expected. The digital technology-friendly environment and budget for digitalization affected the production of positive outcomes. These results are consistent with previous studies reporting the relationship between digitalization and educational background (Al-Zahrani, 2015; Scheerder et al., 2020). We determined that educational background did not significantly affect any outcomes even though the digital technology-friendly environment did. These results imply that it is not general educational background that is important but, rather, an environment that does not make digitization seem difficult, such as education about the Internet. The reason why general educational background influenced gaining positive outcomes from a digitalized society in previous studies (Al-Zahrani, 2015; Scheerder et al., 2020) was that those who have a higher educational background are generally more experienced in digital technologies. Active well-being and an open personality roughly influenced gaining positive outcomes. These factors are related to well-being through the process of actively seeking to resolve mental and physical loads (Keyes et al., 2002; González Gutiérrez et al., 2005; Grant et al., 2009; Sugiura et al., 2015). Thus, these factors may promote digitalization, to maintain quality of life, particularly during the COVID-19 pandemic.

On the other hand, the fact that several personality factors specifically influenced gaining Socialization outcomes implies that there are very different kinds of background factors influencing the development of immaterial outcomes. Our results imply that a personality trait that constructs and maintains social relationships is important. We found that leadership and conscientiousness specifically promoted Socialization, while etiquette specifically suppressed it. Those with higher leadership skills tend to gather people, lead the group, and form reciprocal relationships (Sugiura et al., 2015, 2020, 2021). Similarly, those with higher conscientiousness tend to prioritize maintaining reciprocal relationships (Perugini et al., 2003; Lapierre and Hackett, 2007; Dohmen et al., 2008). The fact that etiquette impaired Socialization even though it is a survival-related personality trait was inconsistent with our prediction. However, etiquette is a survival-related personality trait that does not depend on social relationships as seen in the item, “In everyday life, I take care of myself as much as possible.” Thus, if we suppose those with higher etiquette have an opposite behavioral policy against personality traits prioritizing the construction and maintenance of social relationships, our results were consistent. These previous findings and our results imply that social personality traits are needed for people to adapt to a digitalized society. However, there is another possible interpretation regarding the negative effect of etiquette. This negative effect can be interpreted as resistance to adaptation to social change. Etiquette is related to conformity to social norms. In Japan, those with higher etiquette scores tended to wear a mask more but communicate less, including impersonal communication, according to the social norm (Ding et al., 2022). Considering the characteristics of conformity to social norms, those with higher etiquette scores would be unable to change their lifestyle according to changes in circumstances (i.e., rapid digitalization) because they would try to maintain their original daily life.

### 4.4. Negative outcome and related background factors

The finding that an increase in Loneliness, which was the only negative outcome identified in this study, was influenced by several background factors, similar to the positive outcomes, implied that this negative outcome and other positive outcomes were two sides of the same coin. Similar to the effect of gaining positive outcomes, a digital technology-friendly environment, active well-being, and openness promoted Loneliness. Those with higher digital technology-friendly environment scores would know digital technologies and have more opportunities to use them; therefore, those individuals would recognize negative outcomes more compared with those who have fewer opportunities to use digital technologies. Considering several previous studies pointing out mental and physical loads in digitalized society (Rajani and Chandio, 2004; Collins and Wellman, 2010; Slonje et al., 2013; Alam et al., 2014), we can speculate that the influence of active well-being and openness is related to maintaining a healthy life, which is same as the effect of these factors on positive outcomes. Individuals with lower openness and active well-being, who do not care about making progress and their health, tend to be unable to use digital technologies in a positive manner, which could cause mental or physical problems, such as Internet addiction.

On the other hand, two background personality factors, unfamiliarity with digital technologies and analog preference, were determined to specifically affect an increase in Loneliness. It is not surprising that these factors promoted Loneliness, considering the items. These factors should be ameliorated by improving the digital technology-friendly environment.

### 4.5. Summary of implications

To reveal the kinds of people who gain positive digitalization outcomes, this study established a parsimonious model of outcomes and investigated the relationship between the outcomes and their background factors. As a result, four positive outcomes, including Socialization, Space–time, Economics, and Information, and one negative outcome of Loneliness were identified. The finding that only Socialization was identified as an immaterial outcome implies the importance of self-actualization by online social interactions in gaining immaterial outcomes. Moreover, different background factors influenced each outcome. These results imply the need to intervene with regard to specific background factors, depending on the target outcomes to be improved. Notably, background factors influencing Socialization should be considered. Socialization is the social immaterial outcome that can be gained by a higher level of digitalization; therefore, those with a greater Socialization score adapt and play an active role in a digitalized society. The unique factors influencing gaining Socialization were leadership, etiquette, and conscientiousness, implying the importance of constructing and maintaining social relationships when gaining Socialization (Perugini et al., 2003; Lapierre and Hackett, 2007; Dohmen et al., 2008; Sugiura et al., 2015). On the other hand, gaining Loneliness, a negative outcome was promoted by a lack of a digital-friendly environment.

These findings establish the common concept of digitalization outcomes for digitalization research, in which various outcomes have been used according to the researchers’ interests. Moreover, this study comprehensively examined digitalization-related personality factors, including disaster survival personalities (Sugiura et al., 2015). Thus, our results expand and generalize the relationship between digitalization and personality traits, compared with conventional research focusing on digitalization in the workplace and related personality traits (Diller et al., 2020; Maran et al., 2022; Weber et al., 2022).

We speculated that improvement in the environment by government agencies is a prerequisite, and further humanistic education and intervention to influence these social personality traits is essential. As previous studies have reported, accessibility to digital technologies and education regarding digitalization are fundamental (Gorski, 2005; Scheerder et al., 2020; IDEA, 2022). However, this study showed that improving these environments is insufficient to gain social immaterial outcomes. To ensure a smooth transition to a digitalized society and develop individuals who can play an active role in society, it is necessary to consider two lines of support: the environmental improvement that makes individuals familiar with digital technologies, and the humanistic education that fosters general social skills.

### 4.6. Limitation

This study had several limitations. First, we conducted online surveys; thus, participants were limited to those who knew about using the Internet to make money. We may not have recruited those who could not adapt themselves to a digitalized society. To cover people who cannot adapt to a digitalized society at all, a replication study is needed through an in-person survey. On the other hand, conducting the online survey is one of our strengths because this study certainly recruited people who more or less already adapted to a digitalized society. Second, we did not directly ask about negative outcomes of a digitalized society in the first online survey. However, some participants described the negative outcomes of a digitalized society even though we asked them to describe the positive outcomes. These descriptions of negative outcomes were related to Loneliness; thus, we investigated participants’ thoughts about the negative outcomes of a digitalized society to some extent. Finally, several inconsistencies between our results and previous studies should be discussed. We could not observe several outcomes referred to in previous studies. Participants did not describe skill-building immaterial outcomes in the first free descriptive online survey even though previous studies assumed skill-building immaterial outcomes as one of the outcomes in digitalized society (Rajani and Chandio, 2004; Alam et al., 2014; Scheerder et al., 2020). Our results implied that participants did not regard skill-building outcomes as digitalized society-specific outcomes or participants included skill-building factors into descriptions related to social immaterial outcomes, such as they could see others whom they would not tend to see in everyday life. Previous studies reported several physical negative outcomes, such as decreased physical activity (Rajani and Chandio, 2004; Alam et al., 2014), however, physical problems related to digitalization were not included in the free description survey. We did not ask about negative outcomes in the first free-description survey. Digital technologies for promoting health have become common and several studies have reported the relationship between digitalization and health improvement (Bhavnani et al., 2016; Scheerder et al., 2020). Thus, there is a possibility that physical negative outcomes were no longer regarded as the main negative outcomes. The final inconsistency was the relationship between digitalization and background factors. Previous studies have reported that individuals with higher openness and lower neuroticism tend to digitalize more (Diller et al., 2020; Maran et al., 2022) even though we determined that participants with more openness experienced negative outcomes and that there was no significant neuroticism effect. This previous study focused on digitalization regarding business instead of daily life; however, the aim of this study was that of outcomes of digitalized society, including business and daily life situations. These differences may have caused inconsistencies, implying that different factors underlie digitalization according to the purpose.

## 5. Conclusion

This study established parsimonious models of digitalization outcomes, and relationships between each outcome and background factors. As a result, our hypothesis, that technical-environmental factors commonly influence outcomes while social personality factors particularly facilitate social immaterial outcomes, was supported. We revealed that a prosocial personality (i.e., a personality trait prioritizing construction and maintenance of social relationships), as well as the improvement of technical-environment factors, should be cultivated to help individuals play an active part in digitalized society. Our results showed there were negative outcomes of a digitalized society, outcomes that could be mitigated by improving the environment relating to digital technologies.

## Data availability statement

The raw data supporting the conclusions of this article will be made available by the authors, without undue reservation.

## Ethics statement

The studies involving humans were approved by the Ethical Committee of the International Research Institute of Disaster Science, Tohoku University, Japan. The studies were conducted in accordance with the local legislation and institutional requirements. The participants provided their written informed consent to participate in this study.

## Author contributions

AH, NM, and MS conceptualized this study. YH, AH, NM, and MS contributed to the design. YH and MS interpreted the results. AH, NM, AT-I, KO, and RI contributed and commented regarding analysis methodologies, determination of candidate items of questionnaires, and interpretation of the results. YH performed the statistical analysis and wrote the first draft of the manuscript with support from MS. All authors contributed to manuscript revision and have read, and approved the submitted version.

## Funding

This research was supported by the Grant-in-Aid for Scientific Research on Innovative Areas (Research in a proposed research area; KAKENHI 22H04855) from the Japan Society for the Promotion of Science and Subsidy for Interdisciplinary Study and Research Concerning COVID-19 from the Mitsubishi Foundation (MS).

## Conflict of interest

The authors declare that this study was conducted in the absence of any commercial or financial relationships that could be construed as a potential conflict of interest.

## Publisher’s note

All claims expressed in this article are solely those of the authors and do not necessarily represent those of their affiliated organizations, or those of the publisher, the editors and the reviewers. Any product that may be evaluated in this article, or claim that may be made by its manufacturer, is not guaranteed or endorsed by the publisher.

## Supplementary material

The Supplementary material for this article can be found online at: https://www.frontiersin.org/articles/10.3389/fpsyg.2023.1230192/full#supplementary-material
