# Supplementary material for: Five major outcomes of digitalization: relevance of a survival personality type during COVID-19 pandemic
Source: Front Psychol. 2023 Aug 18;14:1230192. doi: 10.3389/fpsyg.2023.1230192 (PMC10473088; doi:10.3389/fpsyg.2023.1230192)
Supplement: Supplementary file 1 [file Table_1.DOCX]

**Supplementary table 1. Standardized regression coefficients and coefficient of determinations of each step**

|  | **Socialization** | | | **Space-time** | | | **Isolation** | | | **Economic** | | | **Information** | | |
| --- | --- | --- | --- | --- | --- | --- | --- | --- | --- | --- | --- | --- | --- | --- | --- |
|  | **Step1** | **Step2** | **Step3** | **Step1** | **Step2** | **Step3** | **Step1** | **Step2** | **Step3** | **Step1** | **Step2** | **Step3** | **Step1** | **Step2** | **Step3** |
| Age | -3.61 | -2.04 | -2.27 | -2.11 | -1.01 | -1.09 | -1.74 | -1.26 | -1.04 | -1.48 | -1.38 | -1.61 | -0.15 | -0.05 | -0.09 |
| Personal income | 1.50 | -0.41 | -1.02 | 1.35 | -0.62 | -0.62 | -0.49 | -0.33 | -0.42 | 0.91 | -0.57 | -0.51 | 0.02 | -0.41 | -0.43 |
| Educational background | 1.25 | 0.10 | 0.05 | 1.24 | 0.07 | 0.04 | 0.17 | 0.13 | 0.10 | 0.94 | 0.08 | 0.16 | 0.05 | -0.21 | -0.21 |
| Unfamiliarity with digital technologies |  | 0.14 | 0.09 |  |  |  |  | 0.13 | 0.60 |  | -0.17 | -0.18 |  |  |  |
| Analog preference |  |  |  |  |  |  |  | 0.40 | 0.34 |  |  |  |  |  |  |
| Budget for digitalization |  | 0.60 | 0.30 |  | 0.73 | 0.51 |  |  |  |  | 0.73 | 0.52 |  | 0.21 | 0.13 |
| Conservativeness |  |  |  |  |  |  |  |  |  |  |  |  |  |  |  |
| Digital technology-friendly environment |  | 1.61 | 1.18 |  | 1.29 | 1.09 |  | 0.93 | 0.67 |  | 0.49 | 0.38 |  | 0.22 | 0.15 |
| Leadership |  |  | 0.57 |  |  |  |  |  |  |  |  |  |  |  |  |
| Problem solving |  |  |  |  |  |  |  |  |  |  |  |  |  |  | 0.06 |
| Altruism |  |  |  |  |  |  |  |  | 0.23 |  |  |  |  |  |  |
| Stubbornness |  |  |  |  |  |  |  |  | -0.10 |  |  |  |  |  |  |
| Etiquette |  |  | -0.73 |  |  |  |  |  | -0.27 |  |  | 0.40 |  |  |  |
| Emotional regulation |  |  |  |  |  |  |  |  | -0.20 |  |  |  |  |  |  |
| Self-transcendence |  |  | 0.20 |  |  |  |  |  |  |  |  |  |  |  |  |
| Active well-being |  |  | 0.73 |  |  | 0.72 |  |  | 0.59 |  |  | 0.31 |  |  | 0.19 |
| Extraversion |  |  |  |  |  |  |  |  |  |  |  |  |  |  |  |
| Agreeableness |  |  | 0.55 |  |  |  |  |  |  |  |  |  |  |  |  |
| Conscientiousness |  |  | 0.53 |  |  | 0.75 |  |  | 0.77 |  |  | 0.41 |  |  | 0.17 |
| Neuroticism |  |  |  |  |  | 0.48 |  |  |  |  |  | 0.46 |  |  | 0.17 |
| Openness |  |  | 0.51 |  |  | 0.57 |  |  | 0.93 |  |  | 0.97 |  |  | 0.29 |
| R^2^ | 0.09 | 0.28 | 0.37 | 0.05 | 0.23 | 0.29 | 0.02 | 0.25 | 0.31 | 0.04 | 0.18 | 0.25 | 0.002 | 0.11 | 0.20 |
| ΔR^2^ |  | 0.19 | 0.09 |  | 0.18 | 0.06 |  | 0.23 | 0.06 |  | 0.14 | 0.07 |  | 0.11 | 0.09 |

Only significant standardized regression coefficients in each step were described. ΔR^2^ indicates how much fitting was improved compared with a prior step.
